# Exploring the Stability and Electronic Properties of Janus TMCSe Monolayers via DFT Calculations

**DOI:** 10.1021/acsomega.4c10022

**Published:** 2025-01-30

**Authors:** Luis Ángel Campos Ortiz, José Israel Paez Ornelas, Luis Ángel Alvarado Leal, Héctor Noé Fernández Escamilla, Atilano Martinez Huerta, Eduardo Gerardo Pérez Tijerina, Noboru Takeuchi

**Affiliations:** † CICFIM Facultad de Ciencias Físico Matemáticas, Universidad Autónoma de Nuevo León, San Nicolás de los Garza, Nuevo León 66450, México; ‡ Centro de Nanociencias y Nanotecnología, Universidad Nacional Autónoma de México, Apartado Postal 14, Ensenada, Baja California 22800, México

## Abstract

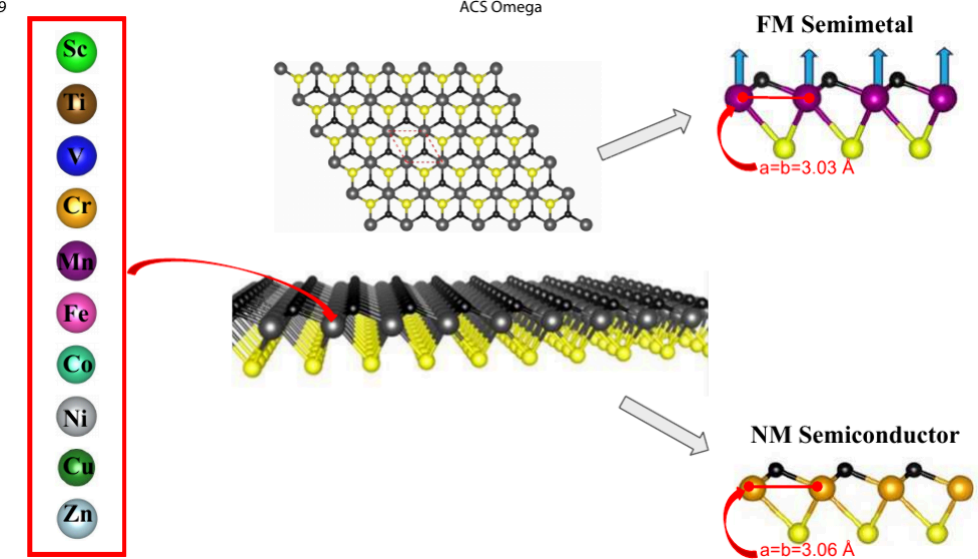

Janus monolayers are two-dimensional materials with distinct chemical compositions on their opposing sides, leading to unique properties and potential applications in various fields. Based on density functional theory (DFT) calculations, we have explored the dynamic stability of a family of Janus monolayers with the general formula TMCSe (TM = Sc, Ti, V, Cr, Mn, Fe, Co, Ni, Cu, Zn). Only two explored systems were dynamically and thermal stable: CrCSe and MnCSe, as evidenced by their phonon dispersion curves and molecular dynamics calculations. Their electronic properties and magnetic character have been investigated using their corresponding density of states. The CrCSe monolayer is a 0.4 eV indirect semiconductor, and the magnetic MnCSe monolayer is spin-up metallic and a spin-down semimetal. Electrostatic potential isosurfaces are used to assess the reactivity of the stable monolayers. They indicate that the surfaces of the TMCSe structures are polarized, with the C side exhibiting a strong negative potential and the Se side displaying a more neutral character. This property may lead to applications in many fields, such as Li storage or toxic molecule trapping.

## 1 Introduction

Since the discovery of graphene by Geim and Novoselov in 2004,^1^ interest in two-dimensional (2D) materials has increased. Different research groups have worked to find new 2D materials with properties comparable to those of graphene. Examples include silicene,^2^ germanene,^3^ transition metal dichalcogenides (TMD),^4−6^ and Janus monolayers.^7^ 2D transition metal dichalcogenides (TMDs) have gained attention due to their unique physical and chemical properties as they transition from multilayers to monolayers. Notable features include the shift from an indirect to a direct band gap,^8^ significant excitonic binding energy,^9^ and the abundance of multiexcitons.^10^ Most TMD monolayers have band gaps in the 1–3 eV range, facilitating the emergence of advanced nanodevices.^11−13^ The presence of an intrinsic direct band gap and the unique electronic and optical properties of 2D TMD monolayers^14^ make them versatile materials for diverse applications as catalysts,^15^ transistors,^16^ components in batteries,^17,18^ and optoelectric applications.^19−22^ TMDs appear in two main phases: honeycomb structures (1H)^23^ (a single layer of the 2H multilayer bulk phase) and centered honeycomb structures (1T).^24^ The structural composition of 2D monolayer transition metal dichalcogenides (TMDs) is characterized by a sandwich-like arrangement comprising a double layer of chalcogen atoms (each on opposing sides) enclosing the transition metal atom. Typically, the chalcogen atoms on both sides correspond to the same chemical element. However, when the chalcogen atoms in the two layers differ, they form a 2D structure known as Janus transition metal dichalcogenide monolayers (JTMDs). These novel materials exhibit asymmetric structures and have garnered considerable attention as a distinct class of 2D semiconductors. Their structural properties render them particularly intriguing for various applications in energy conversion technology,^25^ quantum science,^26^ and the burgeoning field of spintronics.^27^ A methodology for synthesizing JTMD capable of disrupting the out-of-plane structural symmetry has been detailed in ref (28). Furthermore, controlled sulfuration has been employed to synthesize a two-dimensional monolayer with an asymmetric structure known as MoSSe.^29^ Investigation into the Janus MoSSe monolayer has revealed compelling properties for the detection of SO_2_, SO_2_F_2_, H_2_S, SOF_2_, and primarily SOF_6_, showcasing its potential utility in gas-insulated switches (GIS) and the fabrication of ultrahigh sensitivity nanodevices.^30^ Recent studies have also delved into areas such as band gap engineering,^31^ solar water splitting,^32,33^ and photocurrent response,^34^ focusing on the Janus MoSSe monolayer. Additionally, the Janus PtSSe monolayer has been identified for its advantageous properties as a promising photocatalyst, leveraging its internal electric field to facilitate the hydrogen evolution reaction.^35^ Further investigations have been conducted on additional 2D Janus materials, including Janus MXenes^36^ and Janus chalcogenide monolayers of group III.^37^ The band gap of Janus MXenes can be adjusted by selecting a suitable pair of chemical elements to terminate the surfaces and bottom layers of the MXenes.^36^ Janus group-III chalcogenide monolayers exhibit higher piezoelectric coefficients compared to their pristine counterparts.^37^ The unique physical properties of these novel 2D systems, coupled with the successful synthesis of Janus MoSSe monolayers, suggest the potential utility of 2D JTMDs in nanoscale devices for electronic and energy applications.^26^ This study examines a series of Janus TMCSe (TM= Sc, Ti, V, Cr, Mn, Fe, Co, Ni, Cu, Zn) built by a triple single-layer with a TM atom enclosed by C and Se atoms at the opposite faces of the monolayer to explore their dynamic stability and electronic properties further. The C atomic layer was chosen due to its abundance and to explore the difference in electronegativity on the opposite faces of this material. Such a feature is less evident in Janus structures formed by purely chalcogen elements, and the possibility of having a highly polarized surface is desirable for applications in photocatalysis and electronic devices, such as transistors, where asymmetric charge distribution is desirable.^38−40^ This investigation uses first-principles calculations based on density functional theory (DFT). The insights gained from these analyses will inform the future utilization of these monolayers in nanoscale devices.

**Figure 1:**
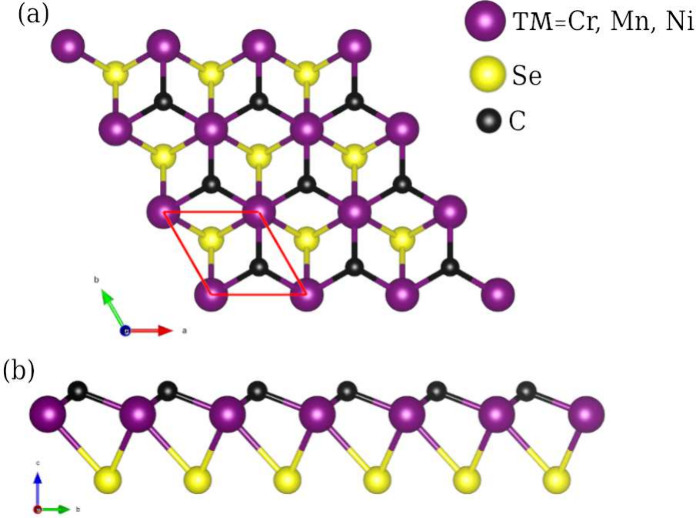
(a) Top view of the Janus TMCSe (TM= Cr, Mn, Ni) monolayer, (b) side view. The continuous red line encloses the unit cell. We used the VESTA^45^ program.

**Table 1:** Optimized Lattice Parameter a, Bond Length, and Bond Angle of the 2D Janus Layers

	lattice parameter (Å)	bond length (Å)		bond angles (deg)
layer	a = b	*d*_*Se*–*TM*_	*d*_*TM*–*C*_	Se–TM–C
CrCSe	3.06	2.5	1.91	86.7
MnCSe	3.03	2.6	1.87	87.3
NiCSe	3.25	2.4	1.93	77.1

**Figure 2:**
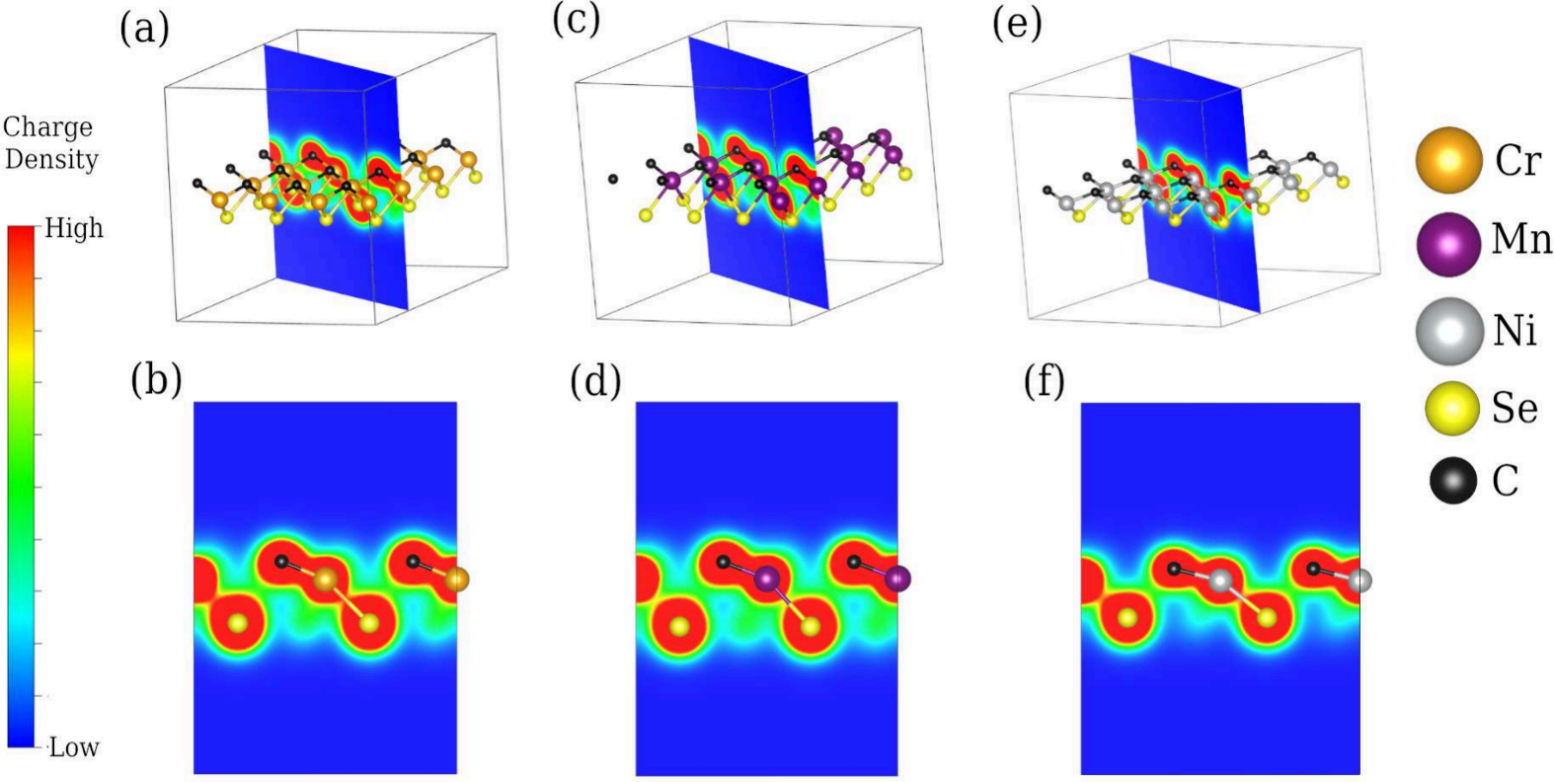
Charge distribution region of Janus TMCSe in (a,b) CrCSe, (c,d) MnCSe, and (e,f) NiCSe. An RGB color code was employed to differentiate charge concentration regions, where blue indicates zero charge and red represents high charge concentration values.

**Figure 3:**
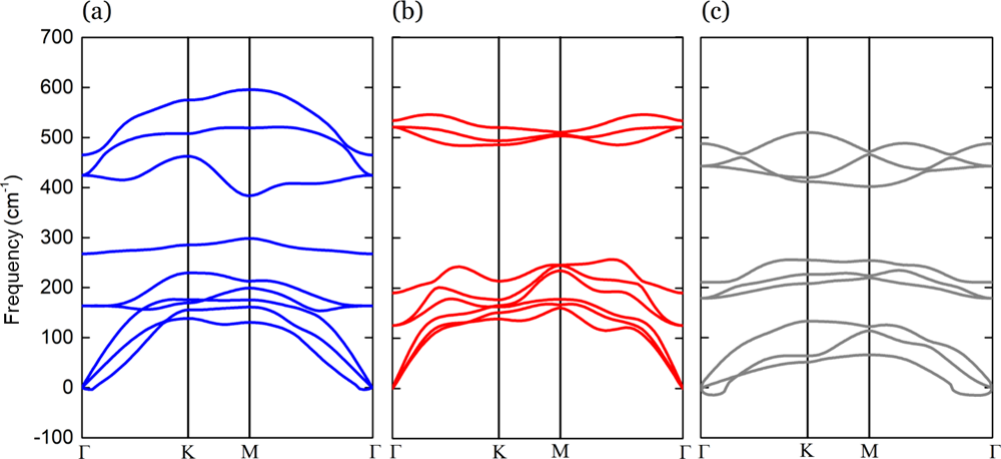
Phonon dispersion analysis of the fully optimized transition metal chalcogenide Janus monolayers: (a) CrCSe, (b) MnCSe, and (c) NiCSe.

## 2 Materials and Methods

Spin-polarized total energy calculations within the density functional theory framework as implemented in the Quantum ESPRESSO package^41^ have been conducted to explore the stability of the proposed systems. The Kohn–Sham states were expanded using a plane wave basis set with a kinetic energy cutoff of 75 Ry and a charge density expansion of 600 Ry. Exchange-correlation energies were treated within the generalized gradient approximation (GGA), employing the Perdew–Burke–Ernzerhof (PBE) functional.^42^ Electron–ion interactions were handled with ultrasoft pseudopotentials.^43^ For Brillouin zone integration,^44^ we have used a Monkhorst–Pack mesh with gamma-centered k-point grids of 8 × 8 × 1 to optimize the Janus layers and 16 × 16 × 1 to calculate electronic properties. An energy convergence criterion was set to 10^–6^ eV. Geometric structures were optimized by minimizing the forces on individual atoms with a convergence criterion requiring all forces on each atom to be smaller than 1 × 10^–3^ Ry/a.u. A vacuum size of 15 Å in the *z*-direction was utilized to prevent self-interactions due to the Born-von Karman boundary conditions.

## 3 Results and Discussion

### 3.1 Structural Properties

To determine the ground state energy of the designed TMCSe monolayers (TM= Sc, Ti, V, Cr, Mn, Fe, Co, Ni, Cu, Zn) through first-principles calculations based on DFT, we started our analysis by conducting a relaxation of the atomic structures by minimizing the Hellmann–Feynman forces^46^ to optimize the lattice constant of the unit cells. However, among all the combinations explored, only three structures exhibited mostly positive phonon scattering bands (see section 3.3). Consequently, in this work, we focus solely on the stable structures CrCSe, MnCSe, and NiCSe (all phonon dispersions are shown in S.I.). Each model possesses a trigonal crystal system (see Figure 1) and belongs to the *P*3*m*1 space group.^47^ The optimized lattice constant of the unit cells was determined by identifying the minimum of the Energy-Volume curve. The lattice constants, bond lengths, and bond angles obtained are shown in Table 1.

### 3.2 Charge Distribution

Motivated by the differences in bond lengths, we examined the variations in bond character associated with each TM and their interaction with C and Se atoms on opposite faces. Figure 2 illustrates a plane projection area that intersects the system and shows the charge distribution regions for the bonds present. An RGB color code differentiates between regions of zero, low, and high charge. The lower part of the figure depicts planes for each system; it can be observed that the TM-C bond on the upper face is consistently characterized by a red color across all models, indicating its strength. In contrast, TM-Se bonds on the lower face are weaker, as denoted by low charge overlap regions, indicating directional anisotropic properties. Note that the TM-Se bond charge distribution is more uniform for Cr and Ni, while Mn exhibits a more localized nature.

### 3.3 Dynamic Stability

To assess the dynamical stability of the predicted 2D materials, we used the Quantum ESPRESSO code to calculate the phonon dispersion path along the high-symmetry points Γ, *K*, and *M* (see Figure 3). The phonon dispersion diagram of each system is composed of nine phonon modes, as the unit cell of the TMCSe monolayers consists of three atoms. The three lower branches correspond to the acoustic phonon modes, while the six upper branches represent the optical phonon modes. Note that for CrCSe and MnCSe(Figure 3(a,b)), a soft crossing between an acoustic and an optical branch is observed, suggesting that the bonds could be prone to geometrical distortions.^48^ The figure illustrates a mode with a small region with small negative values (−4 *cm*^–1^ at the Γ point) for CrCSe, and for NiCSe (−16 *cm*^–1^ at the Γ point) for NiCse. A similar situation has been reported for buckled germanene,^49^ and arsenene,^50^ and it has been attributed to the softening of phonons. As shown in a following section, molecular dynamics simulations show that CrCSe is stable, but NiCSe is not. On the other hand, no imaginary mode is detected for MnCSe. The absence of significant negative modes in the phonon branches across the Brillouin zone implies that all three predicted monolayers are kinetically stable.

### 3.4 Electronic Properties

**Figure 4:**
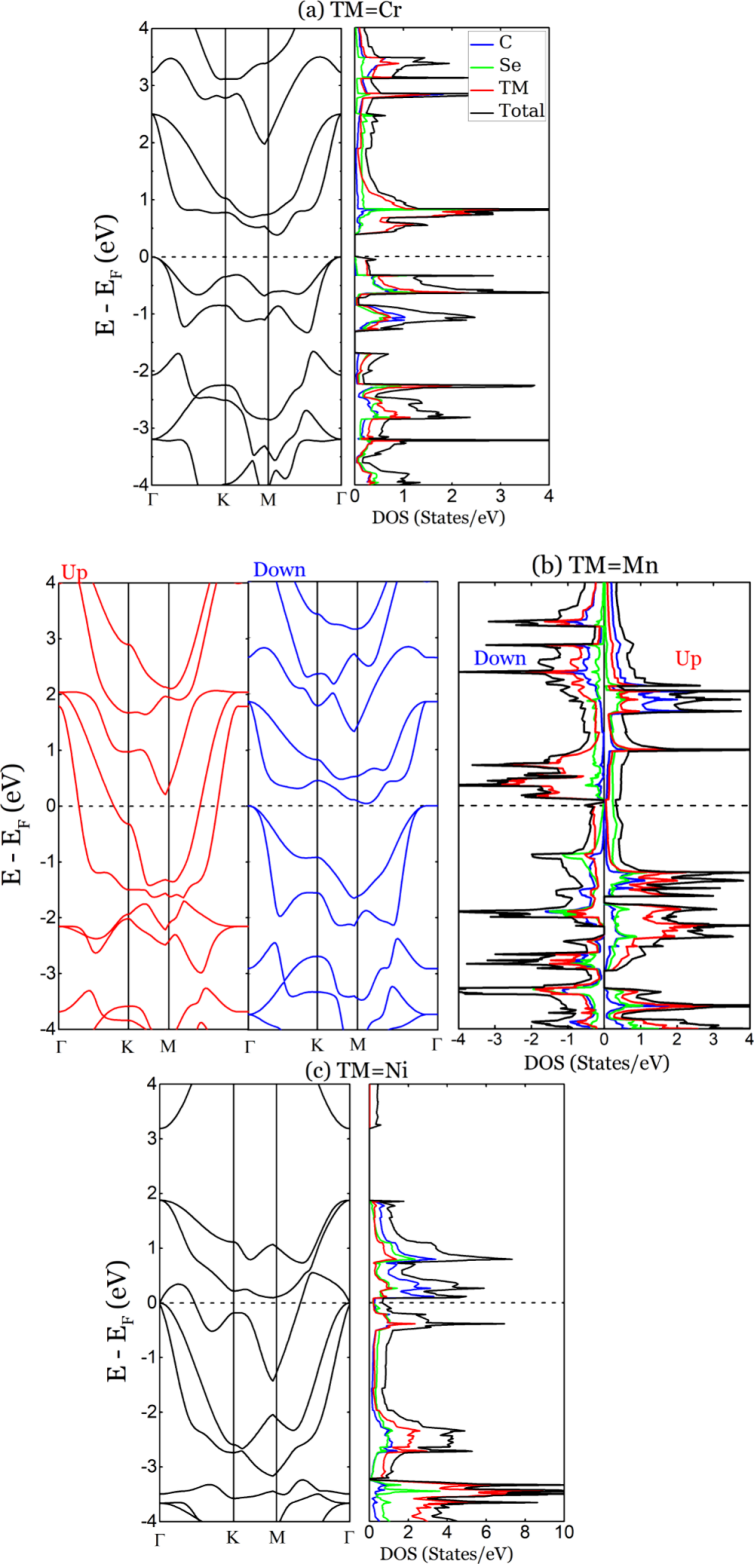
Electronic band structures (black line: spin-unpolarized; red line: spin-up; blue line: spin-down) and density of states of TMCSe. The dashed line denotes the Fermi level, set to zero.

Complementary to the structural characterization, we investigated the electronic properties of the proposed TMCSe monolayers by analyzing their spin-resolved band structures and density of states. The calculated spin-resolved band structures of the TMCSe compounds are displayed in Figure 4. The bands are projected along the path of the high-symmetry points Γ-(0,0,0)-*K*-(0.5,0,0)-*M*-(0.5,0.5,0)-Γ-(0,0,0) to map the first Brillouin zone. The band dispersion indicates that the CrCSe monolayer is a nonmagnetic indirect semiconductor with a band gap of approximately 0.4 eV (see Figure 4(a)). The valence band maximum (VBM) is located at the Γ point, while the conduction band minimum (CBM) is between the *K* and Γ points. The density of states confirms the structure’s semiconducting nature, as there is an absence of states near the Fermi level. The most significant contribution near the Fermi level comes from Cr atoms, whereas C and Se atoms make relatively small contributions compared to Cr. The magnetic MnCSe monolayer presents an intriguing scenario (see Figure 4(b)). The spin-up configuration, exhibits a magnetic metallic behavior; the conduction band crosses the Fermi level at the *K* point, the VBM is located at the Γ point, and the CBM is positioned between the *K* and *M* points. The density of states confirms the metallic nature of the spin-up state of MnCSe, since there is a significant signal near the Fermi level, with the Se making the most significant contribution around the Fermi level, where Mn and C have minimal contributions. In the spin-down configuration, MnCSe also lacks a gap, showing a magnetic semimetal behavior. The VBM is at the Γ point, while the CBM is between the *M* and Γ points. The density of states confirms the metallic nature of the material, with Mn making the most significant contribution around the Fermi level, whereas C and Se have minimal contributions. Finally, the NiCSe monolayer exhibits a nonmagnetic metallic behavior, as depicted in Figure 4(c). The valence and conduction bands intersect near the Fermi level, with the VBM located at the Γ point and the CBM positioned between points *K* and Γ. The density of states confirms the structure’s metallic nature, as there is a significant signal near the Fermi level. The largest contribution near the Fermi level comes from C, although we observe similar contributions from all three elements are observed at negative energies.

**Figure 5:**
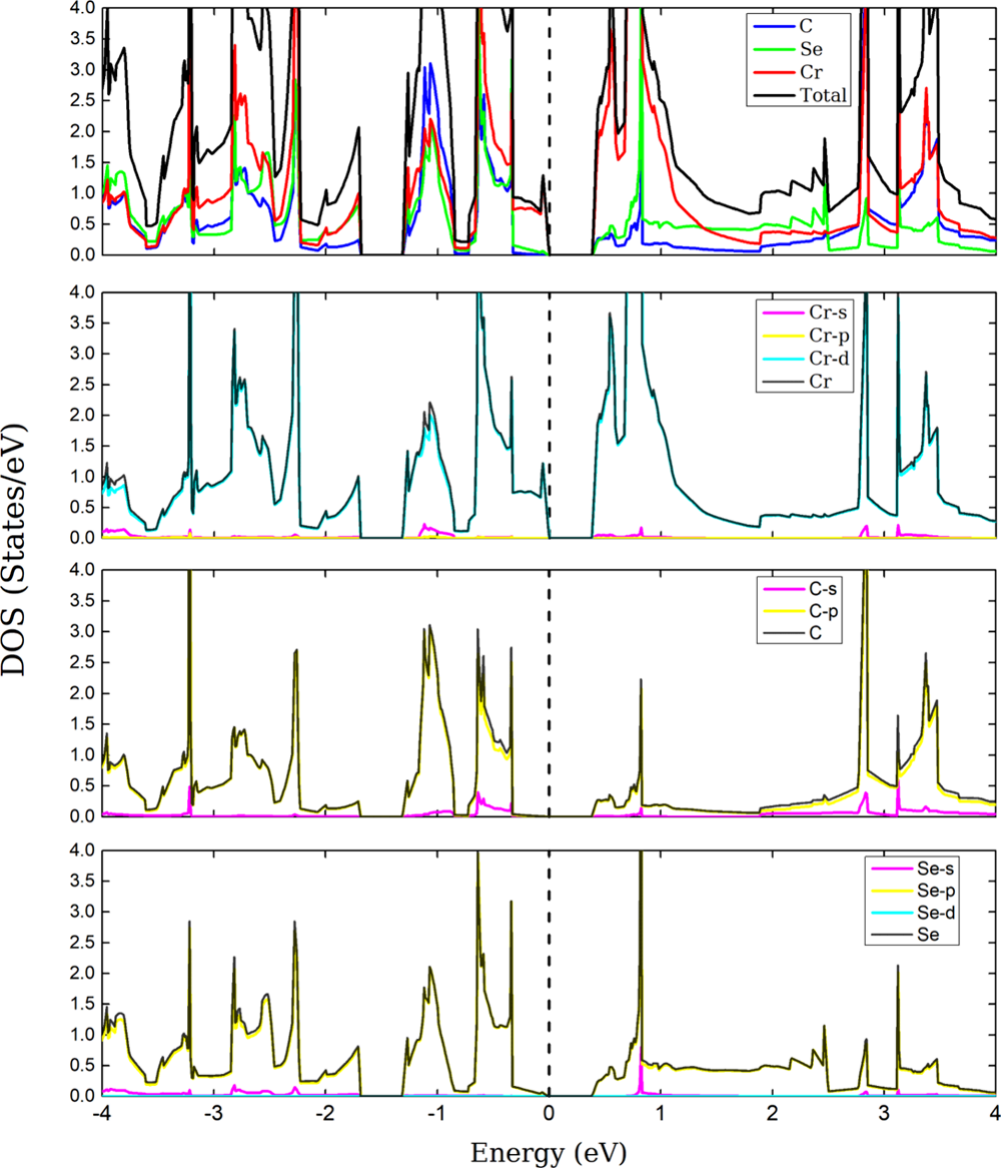
DOS and PDOS of the CrCSe. The dashed line denotes the Fermi level, set to zero.

**Figure 6:**
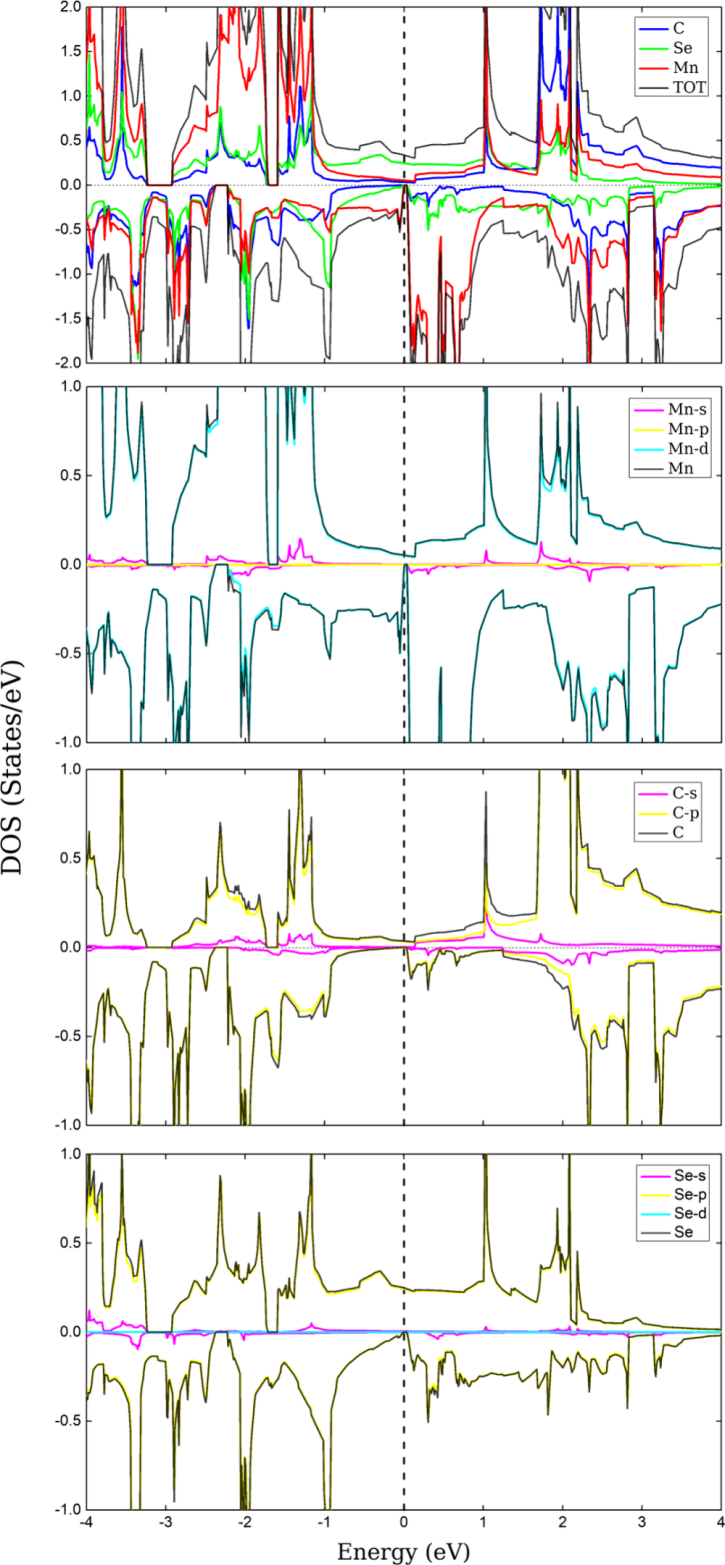
DOS and PDOS of the MnCSe. The dashed line denotes the Fermi level, set to zero.

**Figure 7:**
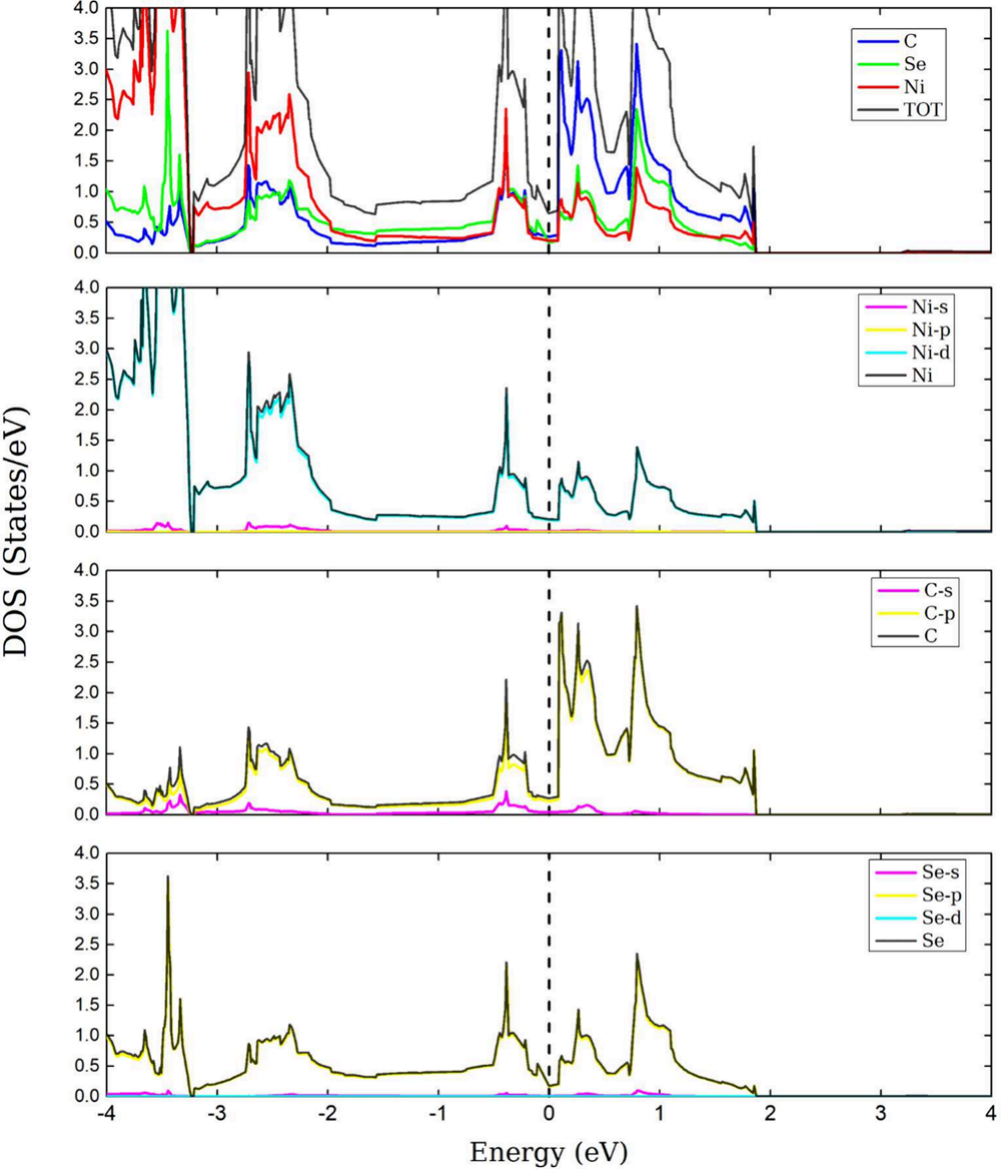
DOS and PDOS of the NiCSe. The dashed line denotes the Fermi level, set to zero.

A PDOS analysis was also performed; in Figure 5, similar signals can be seen near the Fermi level between the d orbital of Cr and the p orbital of C. This indicates hybridization between the atoms. In addition, near 1 eV, a very similar signal is observed between the p orbital of C and the p orbital of Se, thus confirming a hybridization between these atomic species. In Figure 6, a hybridization between the d orbitals of Mn, the p orbital of C, and the p orbital of Se is observed near the Fermi level for the Up state of MnCSe at 1 eV. In the case of C, the s orbital also appears to contribute. Finally, in Figure 7, it is observed that all three atomic species contribute to the metallic state of the monolayer. Hybridization is also seen between the d orbital of Ni, the p orbital of C, and the p orbital of Se near the Fermi level. We observe that there is interaction between atomic species of the three monolayers, which also shows that there is a bond between the atoms.

**Figure 8:**
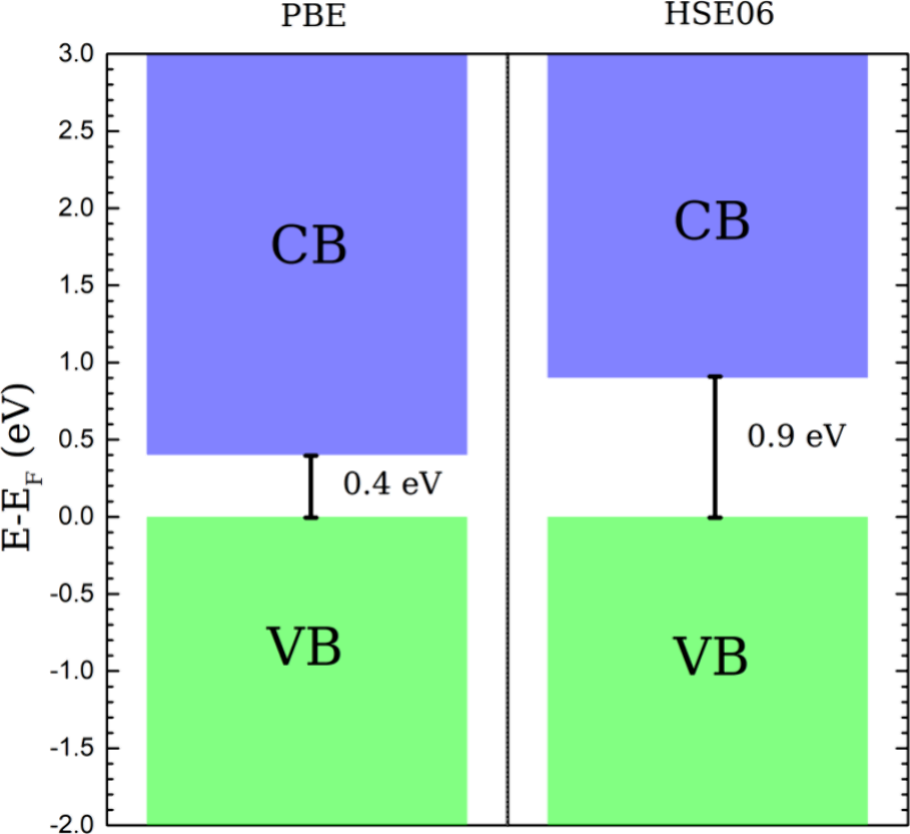
Estimated band gap of the CrCSe monolayer using HSE06 and PBE potentials. The green and blue bars represent VBM and CBM positions, respectively.

The band gap was also recalculated for the semiconductor structure CrCSe using the HSE06 functional. Figure 8 shows an increased band gap of 0.9 eV obtained with this approach compared to the 0.4 eV value obtained with a PBE functional. For the MnCSe and NiCSe monolayers, the HSE06 functional was not employed since the band structure of both systems displays a metallic character with the PBE functional.

### 3.5 Bader Charge Analysis

In this section, we conducted a Bader charge (BC) analysis to investigate the charge transfer and redistribution between the atoms within the TMCSe monolayers. The BC formalism quantifies charge distribution values by atom.^51^ The analysis of charge distribution (Δ*Q* = *e*_*valence*_ – *BC*) in a system can offer valuable insights into electron transfer between atoms, which consequently influences the material’s chemical and electrical properties. Table 2 summarizes the information on the calculated Δ*Q* and bond length values for each system. Our findings reveal that in all three Janus layers, the transition metal acts as an electron donor to the neighboring atoms on the faces of the monolayer, resulting in a positive charge at the center of the sheet and a negative charge on the surface.

**Table 2:** Bader Charge Analysis of the Three Layers

Layer	Atom	**ΔQ(e)**
CrCSe	Cr	1.23
	C	–0.88
	Se	–0.34
MnCSe	Mn	1.07
	C	–0.77
	Se	–0.28
NiCSe	Ni	0.55
	C	–0.44
	Se	–0.11

Notably, in the case of CrCSe, the Cr atom donates more electrons than other transition metals in their respective Janus layers. This observation suggests a stronger interaction between chromium, carbon, and selenium atoms, as indicated in Section 3.2. The BC values help identify the charge transfer mechanism. The fractional values obtained do not correspond to the actual oxidation states of the elements but provide a measure of the charge transfer to and from the atoms

In the system configuration, the TM atoms are located in the interlayer, which is six-fold coordinated, with three C and three Se atoms, resulting in octahedral coordination. In this configuration, crystal field theory for d orbitals predicts that the degeneracy of these orbitals is lifted due to the electrostatic interaction between the metal ion and its surrounding ligands. This interaction leads to different energy levels for the five d orbitals, causing a distortion in their spatial distribution within the crystal field and forming two band groups: the low-energy *t*_2*g*_ and the high-energy *e*_*g*_ bands. Table 2 shows the orbital occupation of the d orbitals for the transition metal atom in the interlayer configuration.

### 3.6 Electrostatic Potential Isosurface

**Figure 9:**
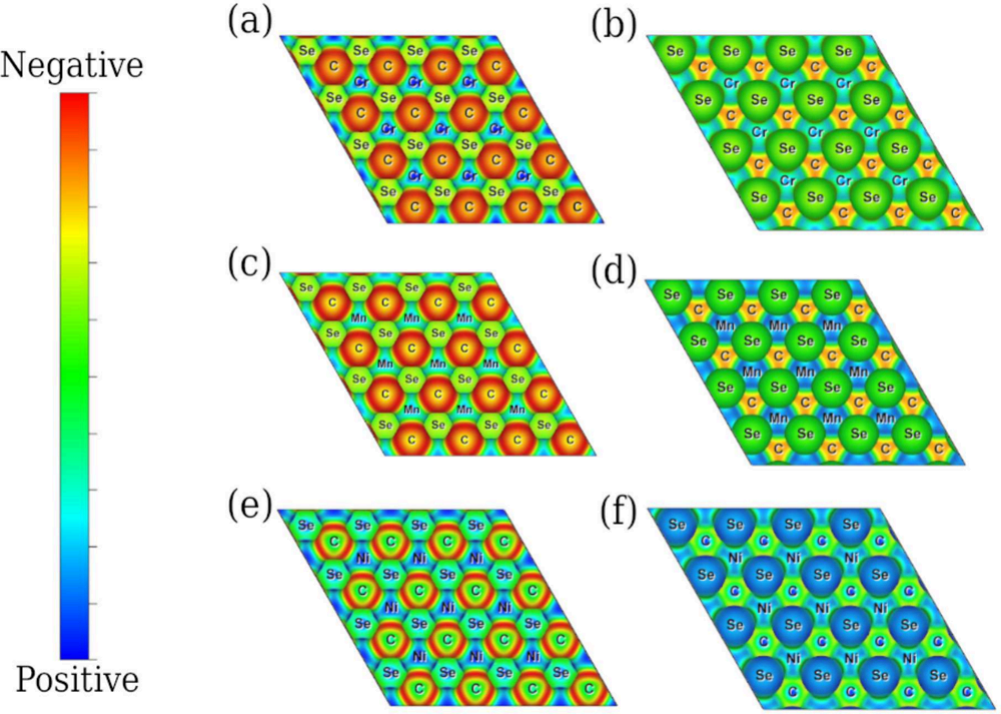
Top and bottom views of the electrostatic potential maps for (a-b) CrCSe, (c-d) MnCSe, and (e-f) NiCSe.

In this section, we analyze the electrostatic potential isosurfaces (EPI) of the TMCSe to identify regions with charge concentrations in real space. These maps offer insights into the distribution of electrostatic charges within the structures, using an RGB color code to represent charge concentration in red and charge depletion in blue. The EPI is depicted using a 0.05 au isovalue. Figure 9 illustrates the EPI of the TMCSe structure. A top view of both faces reveals that the surface exhibits a highly negative potential on the carbon side. Therefore, the C side should show a preferential affinity for molecules with positive potentials. Note that this tendency is maintained for Cr and Mn (see Figure 9(a,c)). However, the C atoms of the Ni system present a less polar character (see Figure 9(e)). Conversely, the Se side of the surfaces displays a green coloring associated with low charge depletion values. The coloring is maintained for Cr and Mn, but again, the Ni system depicts a deeper blue color, indicating a strong charge donor character. Such difference in coloring is consistent with the values obtained through Bader charge analysis. For the Cr and Mn models, the Se captures more electrons (−0.34 and −0.28), while in Ni, it has only −0.11.

The EPI analysis indicates that the surfaces of the TMCSe structures are polarized, with the C side exhibiting a strong negative potential and the Se side displays a more neutral character.

### 3.7 Spin Density

**Figure 10:**
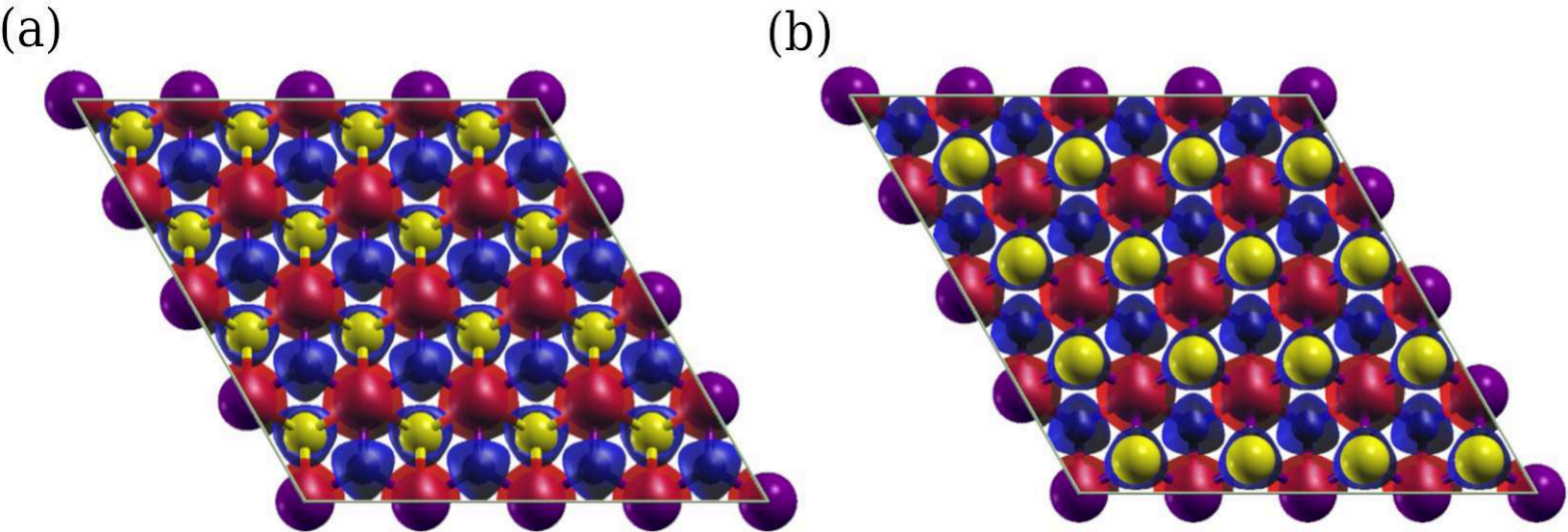
Total spin density distribution for the magnetic system MnCSe (a) top C view and (b) top Se view.

Finally, we examined the total spin density distribution for the magnetic structure MnCSe. The top view of the C (see Figure 10(a)) and Se (see Figure 10(b)) surfaces is presented. Up and down spin contributions are represented in red and blue, respectively. It can be observed that the Mn atoms contribute positively to the spin density, while Se and C exhibit negative contributions to the spin density. This observation agrees with the proposed ionic model, where the Mn atoms donate electrons to the Se and C atoms.

### 3.8 Thermal Stability

**Figure 11:**
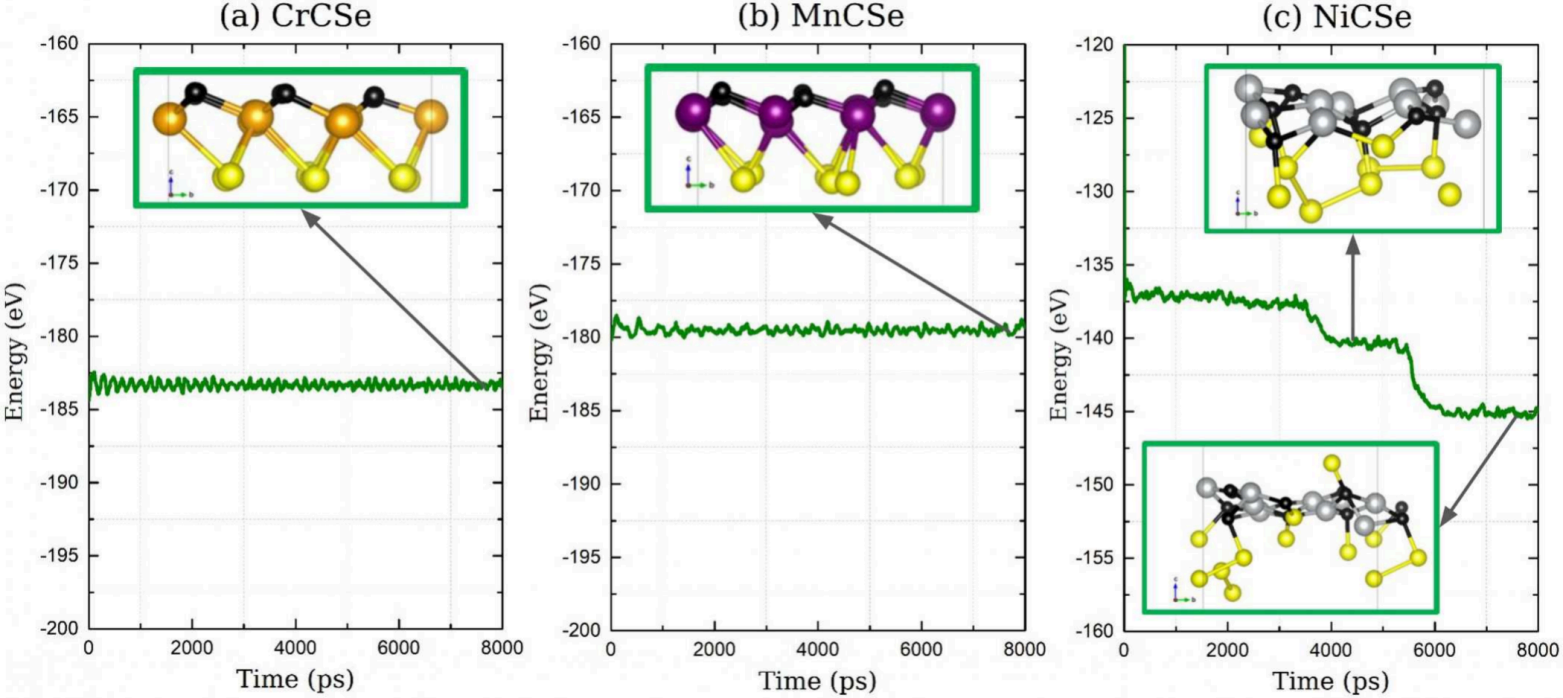
Molecular Dynamic Simulation of the three monolayers, where Energy vs time is plotted.

In this section, the thermal stability of the three Janus monolayers is analyzed using a Molecular Dynamics calculation performed in VASP. To perform the calculation, a temperature of 300 K was set, an NVT assembly was used, and a Nose-Hoover thermostat was employed, setting a simulation time of 8,000 ps. Figure 11(a) shows the CrCSe sheet, which, after 7,500 ps, shows no significant structural deformation; the bonds between C–Cr and Cr–Se are preserved, and the energy value of the monolayer ranges from −183.45 eV to −183.35 eV, demonstrating stability at 300 K. Figure 11(b) shows the MnCSe sheet, which, after 7,500 ps, also shows no significant structural deformation; the bonds between C–Mn and Mn–Se are preserved, and the energy value of the monolayer ranges from −179.31 eV to −179.18 eV, indicating stability at 300 K. Figure 11(c) presents the structure of NiCSe, which maintains its structural integrity for 3,800 ps. After this time, significant structural deformation is observed, which is visible as a decrease in energy. The Ni–Se bonds break, and a different structure is formed, indicating that this monolayer does not demonstrate dynamic stability at 300 K.

## 4 Conclusion

In summary, this study presents findings obtained through first-principles calculations based on density functional theory, proposing two novel Janus monolayers: CrCSe and MnCSe. CrCSe emerges as an indirect semiconductor with a minimal band gap of approximately 0.4 eV. MnCSe exhibits intriguing characteristics, acting as a magnetic metal in its spin-up state while displaying properties of a semimetal in the spin-down state, indicating potential half-metallicity. Structural analyses, including structural search, atomic force minimization, and lattice optimization, confirm that the two monolayers adopt a trigonal crystal structure belonging to the space group *P*3*m*1. Additionally, analysis of their dynamical and thermal stability reveals that both monolayers are stable, as supported by simulated phonon scattering spectra and Molecular Dynamics simulations at room temperature. Furthermore, electrostatic potential maps of the two monolayers suggest their potential utility for ion adsorption, including lithium ions or toxic molecules. These structures also demonstrate promise for applications in emerging electronic devices.

## Data Availability

The data used in the research are all included in the manuscript and the Supporting Information.

## Supporting Information Available

Phonon scattering analysis of all TMCSe optimized monolayers (TM = Sc, Ti, V, Cr, Mn, Fe, Co, Ni, Cu) (PDF)
