# Supplementary material for: Exploring the Stability and Electronic Properties of Janus TMCSe Monolayers via DFT Calculations
Source: ACS Omega. 2025 Jan 30;10(5):4801–8. doi: 10.1021/acsomega.4c10022 (PMC11822503; doi:10.1021/acsomega.4c10022)
Supplement: Supplementary file 1 — ao4c10022_si_001.pdf [file ao4c10022_si_001.pdf]

Supporting Information:

Exploring the Stability and Electronic Properties of Janus TMCSe Monolayers via DFT Calculations

Luis Angel Campos-Ortiz[1], José Israel Paez-Ornelas[2] ∗, Luis Angel Alvarado-Leal[1], Héctor Noe Fernández-Escamilla[1], Atilano Martínez-Huerta [1], Eduardo Gerardo Perez-Tijerina[1], Noboru Takeuchi[2].

[1] CICFIM Facultad de Ciencias Físico Matemáticas, Universidad Autónoma de Nuevo León, San Nicolás de los Garza, Nuevo León, Código Postal 66450, México.
[2] Centro de Nanociencias y Nanotecnología, Universidad Nacional Autónoma de México, Apartado Postal 14, Ensenada Baja California, Código Postal 22800, México.

AUTHOR INFORMATION
Corresponding author
∗E-mail: paez@ens.cnyn.unam.mx (J.I. Paez-Ornelas)

Supporting Information:

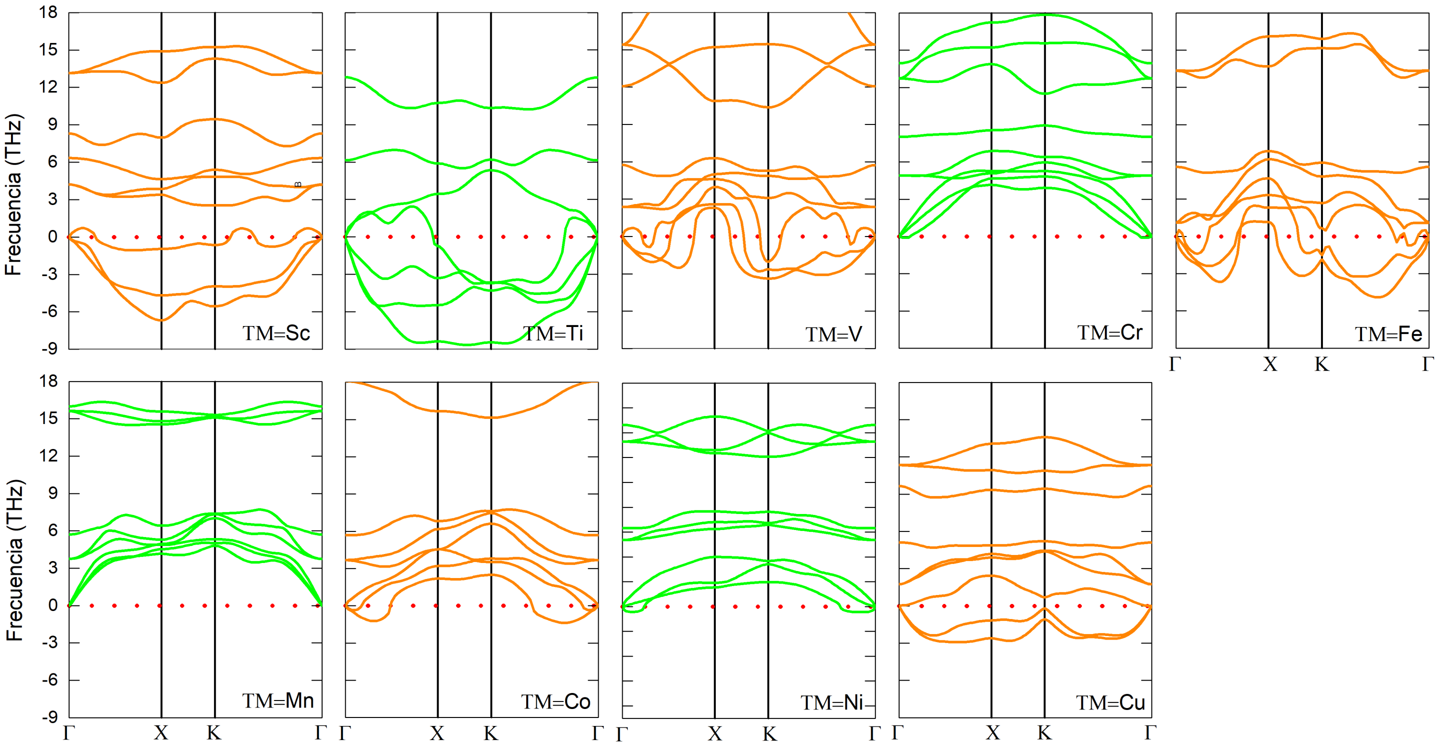

Figure S1: Phonon dispersion analysis of the fully optimized transition metal chalcogenide Janus monolayer.
